# Prevalence of SARS-CoV-2 infection in previously undiagnosed health care workers in New Jersey, at the onset of the U.S. COVID-19 pandemic

**DOI:** 10.1186/s12879-020-05587-2

**Published:** 2020-11-16

**Authors:** Emily S. Barrett, Daniel B. Horton, Jason Roy, Maria Laura Gennaro, Andrew Brooks, Jay Tischfield, Patricia Greenberg, Tracy Andrews, Sugeet Jagpal, Nancy Reilly, Jeffrey L. Carson, Martin J. Blaser, Reynold A. Panettieri

**Affiliations:** 1 Department of Biostatistics and Epidemiology, Rutgers School of Public Health, Piscataway, NJ USA; 2 Environmental and Occupational Health Sciences Institute, Rutgers University, Piscataway, NJ USA; 3 Department of Pediatrics, Rutgers Robert Wood Johnson Medical School, New Brunswick, NJ USA; 4 Rutgers Center for Pharmacoepidemiology and Treatment Science, Health Care Policy and Aging Research, Institute for Health, New Brunswick, NJ USA; 5 Department of Medicine, Public Health Research Institute, New Jersey Medical School, Rutgers University, Newark, NJ USA; 6 RUCDR Infinite Biologics and Human Genetics Institute of NJ and Department of Genetics, Rutgers University, Piscataway, NJ USA; 7 Department of Medicine, Division of General Internal Medicine, Rutgers Robert Wood Johnson Medical School, 125 Paterson Street, New Brunswick, New Jersey 08901 USA; 8 Rutgers Institute for Translational Medicine & Science, New Brunswick, NJ USA; 9 Center for Advanced Biotechnology and Medicine, Rutgers University, Piscataway, NJ USA

**Keywords:** COVID-19, SARS-CoV-2, Healthcare workers

## Abstract

**Background:**

Healthcare workers (HCW) are presumed to be at increased risk of severe acute respiratory syndrome coronavirus-2 (SARS-CoV-2) infection due to occupational exposure to infected patients. However, there has been little epidemiological research to assess these risks.

**Methods:**

We conducted a prospective cohort study of HCW (*n* = 546) and non-healthcare workers (NHCW; *n* = 283) with no known prior SARS-CoV-2 infection who were recruited from a large U.S. university and two affiliated university hospitals. In this cross-sectional analysis of data collected at baseline, we examined SARS-CoV-2 infection status (as determined by presence of SARS-CoV-2 RNA in oropharyngeal swabs) by healthcare worker status and role.

**Results:**

At baseline, 41 (5.0%) of the participants tested positive for SARS-CoV-2 infection, of whom 14 (34.2%) reported symptoms. The prevalence of SARS-CoV-2 infection was higher among HCW (7.3%) than in NHCW (0.4%), representing a 7.0% greater absolute risk (95% confidence interval for risk difference 4.7, 9.3%). The majority of infected HCW (62.5%) were nurses. Positive tests increased across the two weeks of cohort recruitment in line with rising confirmed cases in the hospitals and surrounding counties.

**Conclusions:**

Overall, our results demonstrate that HCW had a higher prevalence of SARS-CoV-2 infection than NHCW. Continued follow-up of this cohort will enable us to monitor infection rates and examine risk factors for transmission.

**Supplementary Information:**

The online version contains supplementary material available at 10.1186/s12879-020-05587-2.

## Background

Healthcare workers (HCW) have emerged as a critical population during the current coronavirus disease-2019 (COVID-19) pandemic. On the frontlines of defense against the virus, HCW may experience increased risk of severe acute respiratory syndrome coronavirus-2 (SARS-CoV-2) infection due to close contact with infected patients [1] and, in many areas, insufficient access to personal protective equipment (PPE) [2]. The plight of HCW during the pandemic has been widely noted [1, 3–7]; as of June 7, 2020, there were over 71,000 confirmed COVID-19 cases (including at least 371 deaths) among HCW in the United States (U.S.) [8].

Our understanding of exposure among U.S. HCW is hindered by several key issues. First, there is clear underreporting of infection in this critical population as CDC data indicate that 84% of all reported U.S. COVID-19 cases had no information on HCW status [9]. Second, among both HCW and non-HCW (NHCW), access to testing has been inconsistent in the U.S., and a large proportion of cases that are asymptomatic or have only mild symptoms are likely to have gone untested [10, 11]. Importantly, asymptomatic or mildly symptomatic individuals can still transmit the virus and may represent the population most likely to spread the infection [12–14]. The rapid spread of the disease and high clinical demands on the HCW population during the pandemic have impaired efforts to prospectively and systematically study the prevalence of SARS-CoV-2 infection in U.S. HCWs. These data are vitally important to understand potential sources of exposure as well as to inform clinical decision-making about staffing and protections for HCW and their patients.

To this end, we report on the baseline prevalence of SARS-CoV-2 infection in previously undiagnosed HCW and NHCW recruited into a prospective observational study conducted within a major university (including an academic medical center) located in New Jersey (NJ), one of the early U.S. epicenters of the pandemic [8].

## Methods

### Design and setting of the study

The Rutgers Corona Cohort (RCC) is a prospective cohort study designed to: (1) characterize factors related to SARS-CoV-2 viral transmission and disease severity in HCW and NHCW; (2) determine the incidence of SARS-CoV-2 and COVID-19 in HCW and NHCW over a six month period. The study was situated at Rutgers University and its affiliated university hospitals, University Hospital (Newark, NJ) and Robert Wood Johnson University Hospital (New Brunswick, NJ). Based on a priori power calculations for the second aim, our goal was to recruit at least 500 HCW and 250 NHCW. For the current analysis, we present cross-sectional baseline data from this ongoing cohort study. The study was approved by the Rutgers Health Sciences Institutional Review Board and all subjects provided electronic written informed consent prior to study activities.

### Study population

**Fig. 1:**
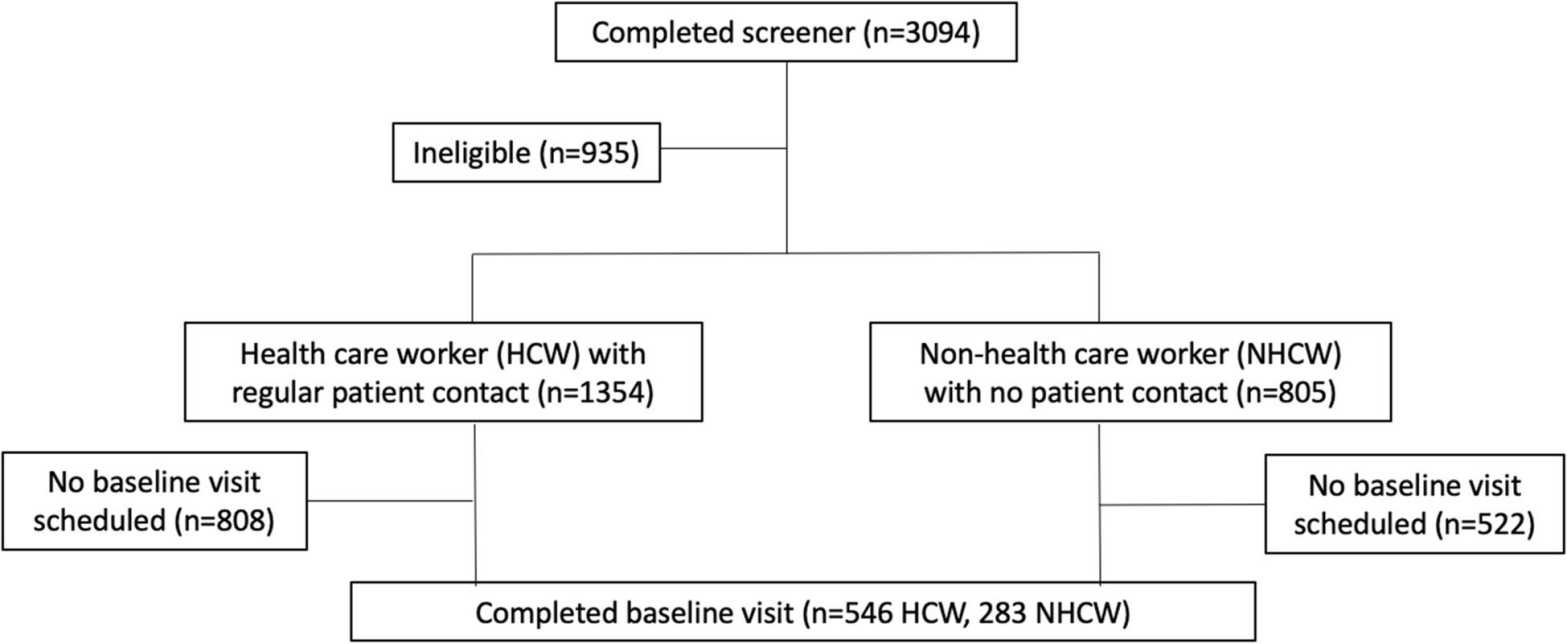
Flow diagram illustrating recruitment into the Rutgers Corona Cohort. Reasons for ineligibility include: pregnancy (*n* = 39), age < 20 (*n* = 45), recent medical treatment or medication change (*n* = 192), recent COVID-19 diagnosis (*n* = 30), insufficient patient contact-HCWs only (*n* = 536), insufficient work hours-NHCWs only (*n* = 93)

From March 24–April 7, 2020, baseline data were collected from RCC participants. Eligible HCW reported: (1) ≥20 h of hospital work weekly; (2) occupations with regular patient exposure (e.g., residents, fellows, attending physicians, dentists, nurse practitioners, physician assistants, registered nurses, technicians, respiratory therapists, physical therapists); and (3) regular direct patient contact (≥3 patients/shift) expected in the next 3 months. Eligibility criteria for NHCW included: (1) faculty, staff, trainees, or students working at Rutgers ≥20 h weekly; and (2) no patient contact. For both groups, additional eligibility criteria were: (1) ≥ age 20; (2) not pregnant or breastfeeding; (3) no urgent care or emergency room visits, hospitalizations, operations, or changes in prescription medicines in the prior 30 days; and (4) no previously diagnosed SARS-CoV-2 infection or COVID-19 (Fig. 1).

### Baseline study activities

An e-mail about the study was sent to all current faculty and staff at Rutgers University (Newark and New Brunswick campuses) and the two participating affiliated hospitals. Interested individuals clicked through a link in the e-mail to access an online pre-screener used to determine eligibility. After informed consent, participants completed an online baseline questionnaire with items on demographics, general health, recent symptoms, lifestyle, occupation, and potential COVID-19 exposure followed by a face-to-face baseline visit. Trained study staff wearing personal protective equipment (PPE) measured body temperature and collected oropharyngeal swabs (OPS). Participants presenting with fever ≥100.4 °F were excluded. Study data were collected and managed using REDCap electronic data capture tools hosted at Rutgers Robert Wood Johnson Medical School [15].

### SARS-CoV-2 assays

Assays were conducted under FDA approved EUA#200090. Following collection, Dacron OPS were immersed in phosphate-buffered saline (PBS) and transported at room temperature to RUCDR Infinite Biologics® (Piscataway, NJ) within 2 h [16]. Total RNA was extracted with Chemagic 360 (PerkinElmer) automation utilizing paramagnetic beads that bind nucleic acids (Chemagic Viral DNA/RNA 300 Kit H96). This system eliminates manual sample handling, reduces risk of cross-contamination and ensures rapid and consistent processing. Reverse transcriptase-PCR (RT-PCR) was performed using the Applied Biosystems TaqPath COVID-19 Combo Kit with 5 μL of the extracted RNA sample. The Rutgers Clinical Genomic Laboratory TaqPath SARS-CoV-2 assay targets by quantitative real-time reverse transcription-PCR (RT-qPCR) three specific genomic regions of the SARS-CoV-2 genome; the nucleocapsid (N) gene, spike protein (S) gene, and ORF1ab region. There are positive and negative assay controls, and MS2 phage is a positive control of nucleic acid extraction and RT-PCR. Assays were performed in triplicate. The lower limit of SARS-CoV-2 detection is 200 copies/mL and the assay exhibits no cross-reactivity with 43 organisms and viruses tested.

Participants who tested positive for SARS-CoV-2 were informed by a study physician, who assessed participants’ clinical condition and provided guidance on medical care, self-isolation, and cleaning [17].

### Statistical analysis

We computed descriptive statistics (i.e., frequencies) of the whole cohort (as well as divided by HCW and NHCW) and used chi-square testing to compare characteristics (demographics, lifestyle factors, sources of exposure) between the two groups. We compared frequencies of positive SARS-CoV-2 test results in relation to participant demographics, social distancing practices, and clinical characteristics using chi-squared tests. Within the HCW group, we further examined frequencies of positive test results in relation to job characteristics (including role, unit, use of PPE, treatment of confirmed or suspected COVID-19 positive patients, and overall patient care). Where applicable for continuous variables, we computed pairwise correlations. We calculated the risk difference of infection between HCW and NHCW groups and obtained 95% confidence intervals for the risk difference using 10,000 nonparametric bootstrap samples. A series of sensitivity analyses were performed examining the risk difference in infection rates between HCW and NHCW after exclusions (for recent symptoms, exposures to confirmed or suspected COVID-19 positive cases outside of work, or both). All analyses were performed using SAS 9.4 and Stata 16.1.

## Results

After advertising across our campuses, 3094 people completed the pre-screening evaluation, of whom 2159 were eligible to participate. Despite the large number of responses, in light of our recruitment goals as well as timing and funding considerations, recruitment was curtailed after 829 subjects (546 HCW and 283 NHCW) had completed baseline visits (Fig. 1). Participants were predominantly female (64.1%), and half (51.6%) were < 40 years old. The cohort was racially diverse (58.3% White, 20.5% Asian, 10.9% Black, 10.4% other/missing), with 12.2% identifying as Hispanic. One-third (34.7%) of participants reported having at least 1 chronic medical condition and 4.5% reported currently smoking. HCW were more likely than NHCW to report having worked on-site at the hospital and/or University (rather than exclusively at home) within the prior week (97.1% vs. 72.1%, respectively), but self-reported social distancing practices (e.g., staying home and avoiding others outside of work) were otherwise similar. Small proportions of HCW and NHCW (12.6 and 7.1%, respectively) reported contact with individuals outside of work with COVID-19 or suggestive symptoms (Table 1).

**Table 1:** Characteristics of the Rutgers Corona Cohort

	Total cohort^**1**^ (*n* = 829)	HCW (*n* = 546; 65.9%)	NHCW (*n* = 283; 34.1%)	*p*-value^2^
**Demographics**				
Sex				0.30
Male	298 (35.9%)	191 (35.0%)	107 (37.8%)	
Female	531 (64.1%)	355 (65.0%)	176 (62.2%)	
Age (years)				0.001
20–39	428 (51.6%)	300 (55.0%)	128 (45.2%)	
40–59	315 (38.0%)	204 (37.4%)	111 (39.2%)	
≥ 60	86 (10.4%)	42 (7.7%)	44 (15.6%)	
Race				< 0.001
White	483 (58.3%)	285 (52.2%)	198 (70.0%)	
Asian	170 (20.5%)	131 (24.2%)	39 (13.8%)	
Black	90 (10.9%)	70 (12.8%)	20 (7.1%)	
Other/missing	86 (10.4%)	60 (1%)	26 (9.2%)	
Hispanic ethnicity	101 (12.2%)	68 (12.5%)	33 (11.7%)	0.74
Current smoker	37 (4.5%)	19 (3.5%)	18 (6.4%)	0.001
**Social distancing**				
Worked on-site (at hospital and/or university) in prior week	734 (88.5%)	530 (97.1%)	204 (72.1%)	< 0.001
Stayed home as much as possible when not working	691 (83.9%)	461 (84.9%)	230 (81.9%)	0.25
Avoided being around other people as much as possible	702 (85.4%)	467 (86.3%)	235 (83.6%)	0.34
Recent exposure outside of work to someone with COVID-19 or new fever, cough, or shortness of breath	88 (10.7%)	68 (12.6%)	20 (7.1%)	0.02
**Clinical characteristics**				
Any chronic comorbidity^3^	288 (34.7%)	184 (33.7%)	104 (36.8%)	0.38
Diabetes mellitus	48 (5.9%)	34 (6.3%)	14 (5.0%)	0.44
Hypertension	125 (15.2%)	80 (14.9%)	45 (15.9%)	0.68
Coronary or cerebrovascular disease or heart failure	20 (2.4%)	15 (2.8%)	5 (1.8%)	0.38
Asthma, COPD, or other chronic lung disease	113 (13.6%)	73 (13.4%)	40 (14.2%)	0.76
Autoimmune disease or reported immunosuppressant use	40 (4.9%)	28 (5.2%)	12 (4.2%)	0.57
COVID-19 symptoms in last week (any)^4^	98 (11.9%)	76 (13.9%)	22 (7.8%)	0.02

COVID-19: coronavirus disease-2019; HCW: healthcare workers; NHCW: non-healthcare workers

1 Percentages may not add up to exactly 100.0 due to rounding

2 Chi-squared tests

3 Chronic comorbidities included diabetes mellitus, hypertension, coronary or cerebrovascular disease, heart failure, asthma, chronic obstructive pulmonary disease, other chronic lung disease, or chronic autoimmune disease

4 COVID-19 symptoms included fever, cough, shortness of breath, vomiting, diarrhea, or change in smell or taste

Overall, 40 HCW (7.3%) and 1 NHCW (0.4%) tested positive for SARS-CoV-2 infection (41/829; 4.9%), representing 7.0% greater absolute risk (95% confidence interval for risk difference 4.7, 9.3%) of SARS-CoV-2 among HCW compared to NHCW. Positive test results were more frequent among participants identifying as Black (8.9%), “other” (9.3%), and Hispanic (11.9%) participants relative to White (3.9%) and Asian participants (3.5%) (Supplementary Table 1). Of the participants who reported having at least one symptom consistent with COVID-19 in the prior week, 17.3% tested positive as did 8% of participants who reported close contact with individuals outside of work who had symptoms or diagnoses of COVID-19 in the last week (Supplementary Table 1).

In total, 71% of all participating HCW reported working with at least one patient per shift who was known or suspected to be COVID-19 positive. HCW who reported recently caring for 5 or more patients with suspected or confirmed COVID-19 were more likely to be positive (24/226, 10.6%) than HCW reporting caring for fewer patients with known or suspected COVID-19 (15/310; 4.8%). Nurses had the highest rate of observed infection (11.1% positive) compared to 1.8% of attending physicians and 3.1% of resident and fellow physicians. Intensive care unit (ICU) workers had low rates of observed infection (2.2%), compared to those working on other units (4.9–9.7%) (Table 2).

**Table 2:** Rates of SARS-CoV-2-infection among healthcare workers at two New Jersey hospitals (Robert Wood Johnson University Hospital [RWJUH] and University Hospital Newark [UHN])

Variable	# SARS-CoV-2 + / total *n* (%) 40/546 (7.3%)	# SARS-CoV-2 + / *n* at RWJUH (%) 10/290 (3.5%)	# SARS-CoV-2 + / *n* at UHN (%) 30/256 (11.7%)
**Health care role**			
Attending physician	2/112 (1.8%)	1/63 (1.6%)	1/49 (2.0%)
Resident or fellow physician	3/98 (3.1%)	2/62 (3.2%)	1/36 (2.8%)
Nurse	25/225 (11.1%)	7/123 (5.7%)	18/102 (17.7%)
Other	10/111 (9.0%)	0/42 (0%)	10/69 (14.5%)
**Primary unit**^**1**^			
Emergency department	20/245 (8.2%)	8/132 (6.1%)	12/113 (10.6%)
Medical floor	9/185 (4.9%)	5/103 (4.9%)	4/82 (4.9%)
Operating room	13/134 (9.7%)	1/53 (1.9%)	12/81 (14.8%)
Intensive care unit^2^	4/192 (2.2%)	1/111 (0.9%)	3/81 (3.7%)
Designated COVID-19 unit	5/63^3^ (7.9%)	1/28 (3.6%)	4/35 (11.4%)
Other unit	14/255 (5.5%)	4/122 (3.3%)	10/133 (7.5%)
**Estimated percentage of work-time spent in patients’ rooms**			
< 25%	11/210 (5.2%)	1/115 (0.9%)	10/95 (10.5%)
25–49%	7/117 (6.0%)	1/65 (1.5%)	6/52 (11.5%)
50–74%	11/116 (9.5%)	6/66 (9.1%)	5/50 (10%)
≥ 75%	11/95 (11.6%)	2/39 (5.1%)	9/56 (16.1%)
Missing	0/8 (0%)	0/5 (0%)	0/3 (0%)
**Estimated percentage of patients for which PPE**^3^ **was used**			
< 25%	4/87 (4.6%)	2/53 (3.8%)	2/34 (5.9%)
25–49%	2/59 (3.5%)	1/32 (3.2%)	1/27 (3.7%)
50–75%	4/61 (6.7%)	1/29 (3.5%)	3/32 (9.7%)
75–99%	4/41 (9.8%)	2/17 (11.8%)	2/24/ (8.3%)
100%	25/238 (10.5%)	4/128 (3.1%)	21/101 (10.1)
Missing	1/60 (1.7%)	0/31 (0%)	1/29 (3.5%)
**Average number of patients with suspected or confirmed COVID-19 per shift**^**4**^			
0	6/148 (4.1%)	2/82 (2.4%)	4/66 (6.1%)
> 0- < 5	9/162 (5.6%)	0/90 (0%)	9/72 (12.5%)
≥ 5	24/226 (10.6%)	8/113 (7.1%)	16/113 (14.2%)
Missing	1/10 (10.0%)	0/5 (0%)	1/5 (20%)

COVID-19 coronavirus disease-2019; PPE personal protective equipment; RWJUH Robert Wood Johnson University Hospital; SARS-CoV-2 severe acute respiratory syndrome coronavirus-2; UHN University Hospital Newark

1 Half (50.4%) of HCW, including 15 (37.5%) of those infected, reported more than one primary unit

2 Included medical, surgical, cardiac, neurocritical, pediatric, and neonatal intensive care units

3 Personal protective equipment referred to wearing gloves, gown, and a mask (surgical or N95)

4 Decimal places allowed in response

Forty-three percent of the HCW reported using PPE during all patient contacts; of those, 10.5% tested positive for SARS-CoV-2. Rates of SARS-CoV-2 infection were slightly higher among workers who spent greater proportions of time in patients’ rooms, reported higher levels of PPE use, and reported exposure to more patients with suspected or diagnosed COVID-19 (Table 2). We observed a weak positive correlation between the number of patients with suspected or diagnosed COVID-19 and the number of patients for whom any mask, gloves, and gown (r = 0.29) or N95 masks, gloves, and gown (r = 0.35) were used.

Nurses and “other” HCW reported spending a greater proportion of their time in patients’ rooms relative to attendings, residents, and fellows. Nurses and “other” workers also reported the highest use of PPE, with over half of nurses reporting use of PPE with 100% of their patients. Over half of the nurses reported caring for more than 5 patients with confirmed or suspected COVID-19 per shift, compared to approximately 1/3 of participants in the other job roles (Supplementary Table 2).

In sensitivity analyses, after exclusion of individuals with symptoms of COVID-19 at baseline and exposure outside of work to someone with COVID-19 (or COVID-19 symptoms), the observed difference in SARS-CoV-2 infection rates between HCW and NHCW remained but were slightly attenuated (Supplementary Table 3).

## Discussion

At baseline, among 829 participants without previous diagnosis of SARS-CoV-2 infection or COVID-19, 7.3% of HCW and 0.4% of NHCW were found to be SARS-CoV-2-positive. These results support the hypothesis of higher current SARS-CoV-2 prevalence in HCW compared with NHCW, a difference that may be attributable to workplace exposures, given the low rate of infection in NHCW. The potential for exposure in the health care setting is further supported by the observation that only 8% of infected participants reported having a contact outside of work with COVID-19 or symptoms.

We observed variation in infection rates by hospital role and job duties. SARS-CoV-2-infected HCW were more likely to be nurses, to spend more time in patients’ rooms, and to have more patients with suspected or confirmed COVID-19. This may indicate nosocomial transmission among higher risk HCW occurring during the early phase of the local pandemic when the primary routes of viral transmission were still unclear and protections were limited. Higher rates of viral infection and/or antibody positivity among higher risk HCWs (e.g., those serving high-risk COVID-19 medical units) have been reported in some [18, 19] but not all studies [20–23]. Similarly, several other studies have examined SARS-CoV-2 infection by HCW role, but results have been variable. Some studies have reported high rates of infection or antibody positivity among clinicians [24] and lower status support roles, such as janitorial staff [18], whereas other studies observe no differences in infection by job role [19, 20]. One other study specifically reported on infections in nurses, noting that, in contrast to our study, none of the 155 nurses sampled tested positive for SARS-Cov-2 antibodies; however, overall antibody positivity among HCW was quite low in that study (11/406; 2.9%) [25]. Considered as a whole, the body of work on SARS-CoV-2 among HCW suggests considerable inter-hospital variation which may be attributable to differences in hospital policies and practices as well as rates of infection in the surrounding communities.

In our study, reported PPE use was positively correlated with number of patients with suspected or confirmed COVID-19, and HCW who reported lower usage of PPE did not appear to have higher rates of infection, suggesting that use of protective measures was proportional to perceived risks of acquiring infection. The more consistent use of PPE among those providing care for suspected or confirmed COVID-19 positive patients may also explain why ICU workers showed low rates of infection (2.2%) compared to other units (4.9–9.7%) despite providing frontline care for confirmed COVID-19 positive patients. However anecdotal reports of variation in access to PPE, reuse of PPE, and types of PPE provided across hospital units and roles suggest the potential for measurement error which may have obscured our ability to detect associations between use of PPE and SARS-CoV-2 infection. Importantly, a study of 420 HCW in Wuhan, China, during the height of the pandemic demonstrated that universal use of PPE along with other protective measures was highly effective and fully protected HCW, such that no virus or antibody positivity was observed [26].

Consistent with disparities observed among the general public [27–29], infection rates were higher among participants who were Black, Latino, and “other” races. Our study is underpowered to assess the root causes of these disparities, and more research is needed to examine whether racial differences in COVID-19 among HCW may be related to their particular roles in the health care setting or are reflective of residence in communities that are more vulnerable to infection.

**Fig. 2:**
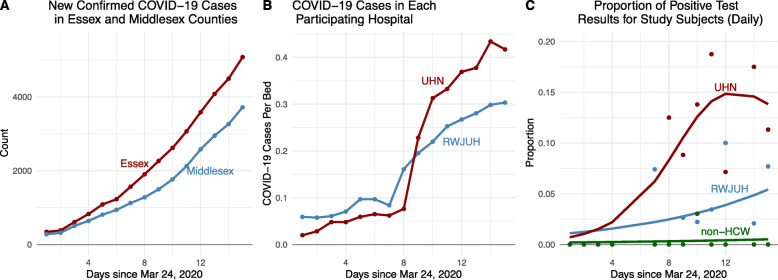
Prevalence of SARS-CoV-2 infection in a prospective cohort, participating hospitals, and surrounding counties during the recruitment period (3/24/2020–4/7/2020). **a** confirmed cases of COVID-19 in Central and Northern New Jersey counties containing the participating hospitals; **b** confirmed inpatient cases of COVID-19 per total hospital beds in participating hospitals; and **c** confirmed SARS-CoV-2 positive cases in healthcare workers (HCW) and non-healthcare workers (NHCW) by hospital in the Rutgers Corona Cohort. County data comes from the New Jersey Department of Health (as reported in the New York Times). RWJUH Robert Wood Johnson University Hospital; SARS-CoV-2 severe acute respiratory syndrome coronavirus-2; UHN University Hospital Newark

Higher rates of current SARS-CoV-2 infections were seen among HCW at the participating hospital with a higher proportion of COVID-19 patients and located in a geographic area with higher infection rates (Fig. 2, Table 2). Over the two-week recruitment period, there was an apparent increase in the number of participating HCW (but not NHCW) testing positive for SARS-CoV-2. This rise was consistent with the sharp increase in confirmed positive cases in the participating hospitals and well as the surrounding areas [30]. With its proximity to New York City (NYC), NJ has been one of the states hardest hit by COVID-19 crisis to date: home to less than 3% of the U.S. population [31], as of July 1, 2020, NJ had over 163,000 confirmed COVID-19 cases, representing 8.5% of all known cases nationwide [8, 30]. At the time our recruitment began on March 24, 2020, 3675 cases had been reported in NJ, second only to New York [30]. Compared to Robert Wood Johnson University Hospital situated in Central NJ, the infection rate among HCW was nearly 3.5 times higher at University Hospital Newark, an urban hospital situated close to NYC with higher population density and higher rates of infections, as well as a higher proportion of hospitalized patients with COVID-19. The difference in infection rates detected among HCW at these two hospitals highlights the variability across hospitals even within the same medical system and the need for more research on COVID-19 in HCW across diverse healthcare settings.

Relatedly, policy and standard practices may differ substantially across hospitals and have evolved rapidly across the pandemic as PPE access, capacity, and understanding of transmission have shifted. At the time of our study recruitment, SARS-CoV-2 testing was quite limited; at both participating hospitals, only symptomatic patients were routinely tested. While attempts were made to separate patients with COVID-19 from others presumed to be SARS-CoV-2-negative, in some cases, COVID-19 patient overflow resulted in mixed units (in negative pressure rooms) at one hospital (University Hospital Newark [UHN]). By contrast, at the other hospital (Robert Wood Johnson University Hospital [RWJUH]), increased capacity to handle the COVID-19 surge allowed for complete separation of COVID-19 units and presumed SARS-CoV-2-negative units. Similarly, there were differences between the hospitals in terms of guidelines for masking: RWJUH enacted different policies for COVID-19 vs. other units; masking policies for clinical care of patients with COVID-19 at UHN depended on the type of procedures being performed (aerosolizing vs. non-aerosolizing). At both hospitals, all staff were provided mandatory training in appropriate donning and doffing PPE. Carefully studying infection transmission in relation to changes in hospital policy and practice will be important moving forward to ensure best practices to protect HCW when future waves of infection occur.

Several studies have reported on the high rates of infections among HCW, including cross-sectional and retrospective studies of symptomatic or hospitalized HCW [3, 5, 7, 32]. Our findings were similar to those of a retrospective cross-sectional study of Chinese healthcare workers, which demonstrated higher case infection rates among HCW (2.1%) compared to NHCW (0.4%), and among the former, higher infection rates among nurses (2.2%) compared to doctors (1.9%) [33]. However, in contrast to that study which relied upon diagnosed cases, our study examined the baseline results from a prospective cohort of HCW without known SARS-CoV-2 infection or COVID-19 diagnosis at the time of screening. Similar work in the United Kingdom is ongoing, with a recent report suggesting that among 400 asymptomatic healthcare workers, up to 7.1% tested positive for SARS-CoV-2 at the height of the London pandemic, with temporal trends mirroring those in the general population [34]. While the parallel trends in infection rates in healthcare workers and the general public may signify community transmission as a greater source of infection than hospital-based exposure, follow-up in our study and others will address that hypothesis and compare rates of SARS-CoV-2 infection between HCW and NHCW. It has been suggested furthermore that most transmission in the healthcare setting occurred at the onset of the pandemic when PPE was extremely limited and before rigorous infection control procedures were implemented. At the same time, there are inherent social distancing challenges in the clinical environment given the multitude of essential activities that cannot be done remotely [35]. Follow-up of our cohort through potential future waves of widespread community infection and COVID-19 hospitalizations will be important to assess these issues [36].

Limitations of this study include rapid recruitment of a convenience sample which may have led to over-enrollment of subjects highly concerned about potential infection. At the time of recruitment, testing in NJ was very limited (even for HCW), and not surprisingly, a large number of people completed the pre-screener, likely motivated by the opportunity to be tested. In our cohort, slightly higher proportions of HCW versus NHCW reported recent COVID-19 symptoms or sick contacts with COVID-19 diagnoses or symptoms, raising the possibility of ascertainment bias. However, recruitment and testing of HCW and NHCW in the same locations and timeline, similar enrollment rates of eligible HCW and NHCW, and the low overall prevalence of recent symptoms or exposures among both HCW and NHCW minimized this source of bias. Importantly, exclusion of participants with prior COVID-19 symptoms or exposures did not change the overall conclusion. Despite biases that could have raised the rates of detected infections, in fact 95% of the cohort were negative for SARS-CoV-2 infection at baseline. Furthermore, as an observational study, we cannot definitively identify the exposures leading to infection. Data on PPE use were limited, self-reported, and did not include specifics on all items used (e.g., eye protection). We cannot rule out HCW infections transmitted from sources other than hospitalized patients, including asymptomatic colleagues or contacts outside the hospital, as other studies have reported [13, 36, 37]. Indeed, the hospital with higher rates of infected HCW had both higher rates of infected patients within the facility as well as higher rates of infections in the surrounding area. Finally, the small numbers of SARS-CoV-2 positive cases at baseline and large number of potentially related factors limited statistical comparisons through multivariable modeling, but longitudinal follow-up of this cohort will provide novel incidence and exposure data as well as greater statistical power to understand factors associated with new infections.

## Conclusions

In summary, in a prospective cohort of individuals previously undiagnosed with SARS-CoV-2, conducted in the early phases of community transmission, the prevalence of active SARS-CoV-2 infection at baseline was considerable higher among HCW as compared to NHCW. We observed higher rates of infection among nurses, in those caring for more patients with suspected or confirmed COVID-19, and in the hospital with a higher proportion of patients with COVID-19. Lower rates of reported PPE use did not appear to correspond to higher rates of infection. Most infected participants reported no symptoms of COVID-19 and had no known sick contacts outside of the workplace. Additional strategies are needed to protect these critical frontline workers and to identify deficiencies in current protections. This prospective HCW cohort provides the baseline data that will be used to study the incidence and other risk factors of SARS-CoV-2 infection in this crucial population as the pandemic continues. Follow-up of this cohort, including serial SARS-CoV-2 viral and antibody testing, is underway.

## Supplementary Information

**Additional file 1:** **Supplementary Table 1.** Rates of SARS-CoV-2 infection in relation to participant characteristics. **Supplementary Table 2.** COVID-19-related job characteristics among health care workers by role. **Supplementary Table 3.** Sensitivity analyses: rates of SARS-CoV-2-infection after exclusions.

## Data Availability

The datasets generated and analyzed for this report are available from the corresponding author on reasonable request.

## Abbreviations

COVID-19: Coronavirus disease-2019
HCW: Healthcare workers
NHCW: Non-healthcare workers
NJ: New Jersey
OPS: Oropharyngeal swabs
PBS: Phosphate-buffered saline
PPE: Personal protective equipment
RCC: Rutgers Corona Cohort
RWJUH: Robert Wood Johnson University Hospital
SARS-CoV-2: Severe acute respiratory syndrome coronavirus-2
UHN: University Hospital Newark
