# Supplementary material for: Prevalence of SARS-CoV-2 infection in previously undiagnosed health care workers in New Jersey, at the onset of the U.S. COVID-19 pandemic
Source: BMC Infect Dis. 2020 Nov 16;20:853. doi: 10.1186/s12879-020-05587-2 (PMC7668027; doi:10.1186/s12879-020-05587-2)
Supplement: Supplementary file 1 — Additional file 1: Supplementary Table 1. Rates of SARS-CoV-2 infection in relation to participant characteristics. Supplementary Table 2. COVID-19-related job characteristics among health care workers by role. Supplementary Table 3. Sensitivity analyses: rates of SARS-CoV-2-infection after exclusions. [file 12879_2020_5587_MOESM1_ESM.docx]

**Supplementary material**

**Prevalence of SARS-CoV-2 infection in previously undiagnosed health care workers in New Jersey, at the onset of the U.S. COVID-19 pandemic**

***Running title: SARS-CoV-2 infection in health care workers***

**Authors:** Emily S. Barrett, PhD; Daniel B. Horton, MD; Jason Roy, PhD; Maria Laura Gennaro, MD, MSc; Andrew Brooks, PhD; Jay Tischfield, PhD; Patricia Greenberg, MS; Tracy Andrews, MS; Sugeet Jagpal, MD; Nancy Reilly, RN, MS; Jeffrey L. Carson, MD; Martin J. Blaser, MD; Reynold A. Panettieri, Jr., MD

**Supplementary Table 1. Rates of SARS-CoV-2 infection in relation to participant characteristics.**

| **Variable** | **# SARS-CoV-2 +/ total n (%)**  **(41/829)** |
| --- | --- |
| **Demographics** |  |
| Sex |  |
| Male | 13/297 (4.4%) |
| Female | 28/531 (5.3%) |
| Age (years) |  |
| 20-39 | 24/428 (5.6%) |
| 40-59 | 12/315 (3.8%) |
| ≥60 | 5/86 (5.8%) |
| Race |  |
| White | 19/483 (3.9%) |
| Asian | 6/170 (3.5%) |
| Black | 8/90 (8.9%) |
| Other/missing | 8/86 (9.3%) |
| Hispanic ethnicity | 12/101 (11.9%) |
| Current smoker |  |
| **Social distancing** |  |
| Worked on-site (at hospital and/or university) in prior week | 40/734 (5.4%) |
| Stayed home as much as possible when not working | 33/681 (4.8%) |
| Avoided being around other people as much as possible | 36/702 (5.1%) |
| Recent exposure outside of work to someone with COVID-19 or new fever, cough, or shortness of breath | 7/88 (8.0%) |
| **Clinical characteristics** |  |
| Any chronic comorbidity^1^ | 17/288 (5.9%) |
| Diabetes mellitus | 2/48 (4.2%) |
| Hypertension | 10/125 (8.0%) |
| Coronary or cerebrovascular disease or heart failure | 3/20 (15.0%) |
| Asthma, COPD, or other chronic lung disease | 5/113 (4.4%) |
| Autoimmune disease or reported immunosuppressant use | 1/40 (2.5%) |
| COVID-19 symptoms in last week (any)^2^ | 17/98 (17.3%) |

COVID-19: coronavirus disease-2019; HCW: healthcare workers; NHCW: non-healthcare workers

1 Chronic comorbidities included diabetes mellitus, hypertension, coronary or cerebrovascular disease, heart failure, asthma, chronic obstructive pulmonary disease, other chronic lung disease, or chronic autoimmune disease

2 COVID-19 symptoms included fever, cough, shortness of breath, vomiting, diarrhea, or change in smell or taste.

**Supplementary Table 2. COVID-19-related job characteristics among health care workers by role.**

|  | **Health care worker (HCW) job role** | | | |
| --- | --- | --- | --- | --- |
|  | Attending physician (n=112) | Resident or fellow physician (n=98) | Nurse (n=225) | Other HCW (n=111) |
| **Estimated percentage of work-time spent in patients' rooms** |  |  |  |  |
| <25% | 63 (56.3) | 59 (60.2) | 44 (19.6) | 44 (39.6) |
| 25-49% | 28 (25.0) | 18 (18.4) | 50 (22.2) | 21 (18.9) |
| 50-74% | 14 (12.5) | 16 (16.3) | 67 (29.8) | 19 (17.1) |
| ≥75% | 7 (6.3) | 3 (3.1) | 61 (27.1) | 24 (21.6) |
| Missing | 0 (0.0) | 2 (2.0) | 3 (1.3) | 3 (2.7) |
| **Estimated percentage of patients for which PPE**^1^ **was used** |  |  |  |  |
| <25% | 28 (25.0) | 25 (25.5) | 21 (9.3) | 13 (11.7) |
| 25-49% | 15 (13.4) | 9 (9.2) | 26 (11.6) | 9 (8.1) |
| 50-75% | 17 (15.2) | 17 (17.3) | 18 (8.0) | 9 (8.1) |
| 75-99% | 13 (11.6) | 5 (5.1) | 15 (6.67) | 8 (7.2) |
| 100% | 35 (31.3) | 32 (32.7) | 119 (52.9) | 52 (46.8) |
| Missing | 4 (3.6) | 10 (10.2) | 26 (11.6) | 20 (18.0) |
| **Average number of patients with suspected or confirmed COVID-19 per shift^2^** |  |  |  |  |
| 0 | 44 (39.3) | 24 (24.5) | 45 (20.0) | 35 (31.5) |
| >0-<5 | 32 (28.6) | 37 (37.8) | 59 (26.2) | 34 (30.6) |
| ≥5 | 36 (32.1) | 35 (35.7) | 117 (52.0) | 38 (34.2) |
| Missing | 0 (0.0) | 2 (2.0) | 4 (1.8) | 4 (3.6) |

COVID-19: coronavirus disease-2019; HCW: healthcare workers; PPE: personal protective equipment

1 Personal protective equipment referred to wearing gloves, gown, and a mask (surgical or N95)

2 Decimal places allowed in response

**Supplementary Table 3. Sensitivity analyses: rates of SARS-CoV-2-infection after exclusions**

| **# SARS-CoV-2+ / total n (%)** | **HCW** | **NHCW** | **Difference (95% CI)** |
| --- | --- | --- | --- |
| Excluding those with recent COVID-19 symptoms | 26/442 (5.6%) | 1/260 (0.4%) | 5.5% (3.2%, 7.8%) |
| Excluding those with recent exposure outside of work to someone with COVID-19 or COVID-19 symptoms | 33/476 (6.9%) | 1/263 (0.4%) | 6.6% (4.2%, 9.0%) |
| Excluding those with recent COVID-19 symptoms or exposure outside of work to someone with COVID-19 or COVID-19 symptoms | 21/416 (5.1%) | 1/245 (0.4%) | 4.6% (2.5%, 7.0%) |

CI: confidence interval; COVID-19: coronavirus disease-2019; HCW: healthcare workers; NHCW: non-healthcare workers; SARS-CoV-2: severe acute respiratory syndrome coronavirus-2
